# A two-month follow-up evaluation testing interventions to limit the emergence and spread of antimicrobial resistant bacteria among Maasai of northern Tanzania

**DOI:** 10.1186/s12879-017-2857-z

**Published:** 2017-12-15

**Authors:** Casey J. Roulette, Mark A. Caudell, Jennifer W. Roulette, Robert J. Quinlan, Marsha B. Quinlan, Murugan Subbiah, Douglas R. Call

**Affiliations:** 1 Department of Anthropology, San Diego State University, 5500 Campanile Drive, San Diego, CA 92182 USA; 2 Paul G. Allen School for Global Animal Health, Washington State University, Pullman, Washington, USA; 3 Department of Anthropology, Washington State University, Pullman, Washington, USA

**Keywords:** Agro-pastoralists, Sub-Saharan Africa, Community-participatory research, Health education and evaluation, Pasteurization

## Abstract

**Background:**

In sub-Saharan Africa, efforts to control antimicrobial resistance (AMR) are aggravated by unregulated drug sales and use, and high connectivity between human, livestock, and wildlife populations. Our previous research indicates that Maasai agropastoralists—who have high exposure to livestock and livestock products and self-administer veterinary antibiotics—harbor antibiotic resistant *Escherichia coli* (*E. coli*). Here, we report the results of a public health intervention project among Maasai aimed at reducing selection and transmission of *E. coli* bacteria.

**Methods:**

Research was conducted in two Maasai communities in Northern Tanzania. Participants were provided with health knowledge and technological innovations to facilitate: 1) the prudent use of veterinary antibiotics (tape measures and dosage charts to calculate livestock weight for more accurate dosage), and, 2) the pasteurization of milk (thermometers), the latter of which was motivated by findings of high levels of resistant *E. coli* in Maasai milk. To determine knowledge retention and intervention adoption, we conducted a two-month follow-up evaluation in the largest of the two communities.

**Results:**

Retention of antimicrobial knowledge was positively associated with retention of bacterial knowledge and, among men, retention of bacterial knowledge was associated with greater wealth. Bacterial and AMR knowledge were not, however, associated with self-reported use of the innovations. Among women, self-reported use of the thermometers was associated with having more children and greater retention of knowledge about the health benefits of the innovations. Whereas 70% of women used their innovations correctly, men performed only 18% of the weight-estimation steps correctly. Men’s correct use was associated with schooling, such that high illiteracy rates remain an important obstacle to the dissemination and diffusion of weight-estimation materials.

**Conclusion:**

Our results indicate that dietary preferences for unboiled milk, concerns over child health, and a desire to improve the health of livestock are important cultural values that need to be incorporated in future AMR-prevention interventions that target Maasai populations. More generally, these findings inform future community-health interventions to limit AMR.

**Electronic supplementary material:**

The online version of this article (10.1186/s12879-017-2857-z) contains supplementary material, which is available to authorized users.

## Background

Efforts to curb the emergence and transmission of antimicrobial resistant (AMR) bacteria within low-income countries are challenged by unregulated access to over-the-counter antibiotics, and close proximity of human-livestock populations [1–3]. Here, we report results of an ongoing community-health project conducted in collaboration with Maasai agro-pastoralists in Tanzania to reduce selection pressures for AMR [4]. To reduce these pressures, we provided Maasai individuals with health messages to increase awareness and knowledge of bacteria and AMR, and training and materials for two technological innovations to facilitate the prudent use of veterinary antibiotics: (1) tape measures and dosage charts to estimate dosage based on livestock body size; and (2) thermometers to enable milk pasteurization. A two-month follow-up evaluation was conducted to determine retention of the health messages and innovation use.

### Antibiotics and antimicrobial resistance in low-income countries

Whereas AMR is an emerging issue globally, it is particularly worrisome in low- and middle-income countries (LMICs), such as in sub-Saharan Africa (SSA), where antibiotics are often less regulated and compliance with best practices is complicated by low literacy and intermittent access to professional care [1]. In SSA, the health costs associated with AMR are elevated by the presence of communicable diseases (e.g., tuberculosis, malaria) that collectively claim more lives than any other cause of death. Almost all of these diseases exhibit resistance to antimicrobial treatments [5]. Further, the close proximity between human and livestock populations elevates the risk of zoonotic pathogens, which can also exhibit resistance [1]. In SSA, for example, over 600 million individuals live in households that keep livestock [6] . Zoonotic diseases can be transmitted through consumption of contaminated livestock products (milk, meat, eggs) and contact with animals or animal byproducts (feces) [7]. In LMICs, limited government oversight of food processing and unregulated, informal, small-scale food production and distribution systems likely increase the risk of consuming contaminated products [1]. Moreover, population-specific cultural beliefs and practices such as livestock handling and milking practices, differences in political-economy including socioeconomic status and urbanization, and demographic attributes including homestead structure, and livestock herd composition, may also increase AMR selection pressures [4].

Issues of AMR in LMICs stress the need for community-level interventions. Public health models emphasize interventions that are both collaborative and culturally-situated [8–11]. The intervention aimed to decrease selection pressures and transmission risk for AMR and support positive health outcomes within Maasai communities by providing knowledge of AMR and innovations to facilitate pasteurization and correct antibiotic dosage.

### The community: The Maasai

Our health intervention emerged as part of an ongoing (since 2012) research collaboration with Maasai communities in Tanzania. The Maasai and related *Maa*-speaking pastoralists are found throughout Tanzania and Kenya. Today, most Maasai are agro-pastoralists, although livestock products still provide the majority of caloric energy. Maasai live in extended family compounds structured by patrilocal residence, and polygyny remains common. Beginning in early adolescence, the division of labor becomes gender specific with females milking cows, preparing family meals, and gathering firewood [12, 13]. Men spend their time tending to livestock and supervising herd management. Some men travel for other, limited business ventures including working in Tanzanite mines and as security guards for safari companies and banks [14, 15]. A small segment of the population includes successful businessmen who have cinderblock houses and/or pit toilets on their compound, and fewer have vehicles.

### Maasai, milking and AMR

Dairy products play a prominent role in Maasai diets, economies, and traditional ceremonies [16, 17]. As with other East African pastoralists, cattle are primarily seen as milk producers and not as regular sources of meat or trade [17]. Dairy products (e.g., butter) contribute between a third and half of the energy in Maasai diets [18]. Cows are normally milked two times a day, primarily by women. Milk is consumed raw, fermented into sour milk, or boiled (either for consumption or to make butter, oil, and cheese-like products). Raw milk is sometimes stored unrefrigerated for up to 12 h, although it is typically consumed earlier, while sour milk can be stored for up to one week [4].

When Maasai boil their milk, they usually do it to “kill diseases” that are present in raw milk and that might make people sick. Despite these perceived health benefits, only 63% of Maasai households in our sample heat-treat their milk prior to consumption [4], in part because collecting firewood is laborious and sometimes dangerous due to the presence of wild animals (as our participants mentioned). Dietary preferences and attitudes regarding “raw” versus “boiled” milk might also account for low rates of heat-treatment. Raw milk, according to our Maasai adult participants, has “full ingredients”, is “thick”, and helps build the body and give energy. In contrast, boiled milk is thin “like water”, lacks full ingredients, and causes constipation. Milk intended for sour milk or butter is typically not heat-treated as the Maasai believe it delays the fermentation process and produces large, hard and unpalatable curds.

While not all Maasai households boil milk, most participants are aware of the importance of hygienic milking principles (see also [19]). Women and girls clean traditional calabash containers (called *engoti* when small and *oloti* when big) that are used for milk collection and drinking. The containers (which are made of gourds called *oldulet*) are rinsed with water or cow urine and cleaned with brushes (called *esosian*) made of the roots of *olmukatan* (*Albizia anthelmintica* Brong. [Fabaceae]) or *oiti* (*Acacia mellifera* [M. Vahl] Benth. [Leguminosae]). Calabashes are then dried and “disinfected” with coals of *oloirien* (*Olea Africana* Mill. [Oleaceae]), a sacred tree [16]. More recently, plastic and metal containers are used. In contrast to traditional calabashes, plastic and metal containers are often rinsed only with water or cleaned with a rag or sisal brush and laundry soap, if available. Examination of milk from containers among Maasai producers, middlemen, dairies, and retailers found that milk from Maasai gourds had the lowest total bacterial count (TBC) while milk from plastic and metal containers had the highest [19].

Consuming raw, contaminated, milk and milk products increases the risk of infection, resulting in more frequent antimicrobial treatment and thereby greater selection for AMR. Consumption of raw milk may also transmit resistant microorganisms directly if the milk itself contains these microorganisms even if not pathogenic per se. AMR commensal bacteria might pose a risk of transmitting resistance traits to pathogenic bacteria through horizontal gene transfer (e.g., plasmid transmission) [20, 21]. Promotion of hygienic milking practices, such as pasteurization or boiling, might therefore reduce selection pressures for AMR. In the course of our qualitative, ethnographic survey, Maasai people expressed a desire for an alternative to boiling milk that did not alter the ingredients, consistency, and palatability of milk as much as boiling. Maasai believe that boiled milk lacks full nutrients and causes constipation. In fact, Maasai requests for an alternative to boiling motivated our pasteurization efforts reported here. Given that pasteurization, relative to boiling, has little effect on the physical characteristics of milk, we expect pasteurization to be viewed more positively than boiling, thus increasing the incidence of heat-treatment.

### Maasai veterinary antimicrobial usage and AMR

Veterinary antimicrobial medicines, what the Maasai call “Swahili” or foreign medicines, are eagerly integrated in Maasai communities. Maasai prefer to use foreign medicine over traditional herbal remedies, in large part because foreign medicines are seen as a more effective treatment for diseases and because they can be easier to obtain than traditional plant medicines that must be foraged from the environment [22]. Although Maasai have adopted foreign medicines, few Maasai have adopted the biomedical model of disease (i.e., germ theory) on which antibiotic medicines are based. Instead, Maasai use of antibiotics is structured by traditional ethnoveterinary knowledge (EVK) [23–26]. Maasai diagnose diseases primarily based upon symptoms, such as piloerection, panting, lethargy, or loss of appetite, but also consider known vectors of disease, season of infection, the species, age, and sex of the impacted animal, recent areas of forage, and diseases that have recently impacted other local herds (26). Diagnosis and treatment are carried out by Maasai men, frequently in consultation with other men.

Our research found that >95% of Maasai households self-administer antibiotics to their livestock while 75% of these households do so without consulting professional veterinarians or livestock officers. These practices underscore the need to ensure prudent antibiotic usage. Injectable oxytetracycline (OTC) (10% and 20% formulations) is the most commonly used antibiotic. If an animal does not respond to treatment within two to three days, then the owner may increase the dosage, switch to a higher concentration, or try a different treatment. Incorrect dosing, which globally is one of the biggest contributors to AMR [5], likely represents a risk factors for the development of antibiotic resistance in Maasai communities. In one of the few observational studies of Maasai use of foreign veterinary medicines, for example, Roderick [27] found that Maasai often administered drugs at dosage rates higher than manufacturer recommendations. In our study communities, Maasai men reported administering the same antibiotic dosage regardless of an animal’s weight. Dosing is problematic because scales are not available and it is difficult to estimate an animal’s weight via visual inspection. In qualitative, ethnographic, interviews, many Maasai men expressed concerns about under- and overdosing livestock, and several explicitly requested our help in determining correct dosages. Those results lead to the specific innovations reported here.

## Methods

### Community interventions

This community-health intervention was situated within a larger National Science Foundation (NSF) funded project investigating the emergence and spread of AMR in the greater Serengeti area. Our first aim was therefore to provide information about the larger NSF project to participating Maasai communities in northern Tanzania, to disseminate knowledge about bacteria, antimicrobial use, and antimicrobial resistance within the context of Maasai veterinary care and milk production, and to provide innovations and instructions to reduce selection pressures and transmission pathways for AMR.

We held a total of six town-hall meetings with 102 women and over 60 men across two villages —Nadonjukin, a village in Simanjiro Region, and Monduli, a village in Arusha region. During these meetings, we presented public health messages regarding the potential for contaminated milk to cause diseases and Maasai difficulties with calculating livestock weight for proper dosing. These messages were situated within the broader context of bacteria and AMR.

To promote pasteurization, we provided thermometers because pasteurization (70 °C for ≥15 s) cannot be observed directly like boiling. The pasteurization procedures were first pilot tested with a small group of women from one homestead. To promote correct antibiotic dosage, we provided measuring tapes to calculate livestock weight and charts to calculate dosage based upon weight. At the meetings, we provided both verbal instructions and direct demonstrations about correct pasteurization and weight estimation for dosage. (See Additional file 1 for an example of the script of the public-health meetings, protocols for using the pasteurization and weight-estimation materials, and images of the meetings, innovations and materials, and training sessions). The Washington State University Institutional Review board and Tanzania National Institute for Medical Research approved our study procedures.

### Evaluation of participant knowledge and innovation use

A major aim of this study is to determine the effectiveness of the intervention to provide insight for future community-health interventions. Specifically, we sought to evaluate patterns of innovation use and retention of public health knowledge in relation to demographic and socioeconomic variables. To this end, two months after the town-hall meetings and dissemination of pasteurization and weight estimation materials, a subset of participants in the largest of the two study communities was located and surveyed for knowledge and skill retention rates and to assess adoption and use of the innovations.

Knowledge retention was assessed using open-ended questionnaires. Using key messages highlighted in town hall meetings, we generated questions to assess knowledge in three domains: 1) bacteria, 2) antibiotic resistance, and, 3) the health benefits of using the innovations (Table 1; for correspondence between the community-meeting script and bacteria and antibiotic resistance knowledge items see in Additional file 2: Table S1).

**Table 1:** Three Domains of Health Messages and Their Associated Knowledge Items

Bacteria	AMR	Health Benefits	
		Thermometers	Measuring Tapes and Dosing Chart
i. There is a variety of different kinds of bacteria	i. Caused by improper use of antibiotics	i. Helps kill bacteria and prevent AMR in milk	i. Helps to calculate weight of animal
ii. Some bacteria can cause diseases	ii. Can be transmitted from animal to human	ii. Provides milk with “full” ingredients	ii. Helps determine proper dose to administer to an animal
iii. They can be transmitted	iii. Diseases resistant to medicines are difficult to treat		iii. Helps prevent the development of AMR
iv. Heat-treatment kills bacteria in milk			iv. It helps improve livestock health

Correct use of the innovations was assessed using behavioral observations in which participants were asked to demonstrate their use of the innovation. During these demonstrations, researchers noted the number of steps performed correctly. Use of the thermometers was evaluated based on three steps: 1) proper placement of the thermometer, 2) knowing the correct temperature for pasteurization, and, 3) holding the temperature for 30 s.^1^ Women were asked to demonstrate or verbally explain each of these steps with the aid of a thermometer. Use of the weight estimation material was evaluated based on six steps: 1) use of the correct (cm) side of the measuring tape,^2^ proper placement of the measuring tape for measuring 2) length, and 3) girth, 4) identifies length, and girth on the dosing chart, 5) finds corresponding weight on dosing chart, and 6) calculates proper dose from the weight. Men were asked to demonstrate these steps on small livestock residing in or near their homestead (for the full questionnaire, see in Additional file 2: Table S2).

### Analysis

Descriptive statistics were used to describe knowledge/skill retention and adoption of innovation behaviors. To examine the association between these outcomes and demographic and socioeconomic variables, generalized linear models were specified. Poisson models were used to identify the factors related to the correct number of steps performed and number of AMR and bacteria knowledge items recalled. Results of Poisson models are interpreted as incidence rate ratios. Logit models were used to model the factors related to whether an innovation was used (1) or not (0). Logit model results are interpreted as odds ratios. Models included relevant knowledge/skill retention factors to determine if our intervention efforts were correlated with retention or adoption. To identify these factors a backward stepwise regression with a retention level of 0.20 was used. We note the significant results of our reduced models in the main body of the text (i.e., *P* < .05) while full models are available in Additional file 3.

To examine model fit, we compare full and reduced models across a range of indices. Bayesian Information Criteria differences between full and reduced models indicated strong or very strong support for better model fit. All model fit indices are provided in Additional file 3. Global fit of reduced models was examined through log likelihood chi-square tests. With the exception of reduced models not containing significant predictors, all chi-square tests indicated good overall fit. Variance inflation factors (VIF) were calculated for each reduced model to assess issues of multicollinearity (see Additional file 3). For all models, mean VIF was below or slightly above 2.00 and no individual predictors were above 4.00. VIFs for every model are provided in Additional file 3.

## Results

### Summary statistics

Summary demographic and socioeconomic statistics for the subset of Maasai participating in the follow-up study are reported in Table 2.

**Table 2:** Summary Demographic and Socioeconomic Statistics^a^

	Women	Men
N	42	23
Age^b^	32.68 (13.06)	45.43 (12.41)
Education (1 = yes 0 = no)	0.26 (0.45)	0.35 (0.48)
Highest class completed	6.45 (1.03)	5.88 (1.96)
Married (1 = yes 0 = no)	1.00 (0.00)	1.00 (0.00)
Number of wives/cowives	1.90 (1.91)	2.22 (1.20)
Have children (1 = yes 0 = no)	0.98 (0.15)	0.96 (0.21)
Number of children^a^	4.52 (2.90)	9.57 (8.06)
Cattle	27.71 (52.27)	82.34 (148.14)
Cinder block house/pit toilet	0.24 (0.43)	0.13 (0.34)

^a^Highest class completed ranged from Grade 3 to Grade 7. Statistics reported are the mean and standard deviation in parentheses

^b^N = 64, 42 women and 23 men. One women’s self-reported age is missing

### Knowledge of bacteria, AMR, and health benefits of the innovations

**Fig. 1:**
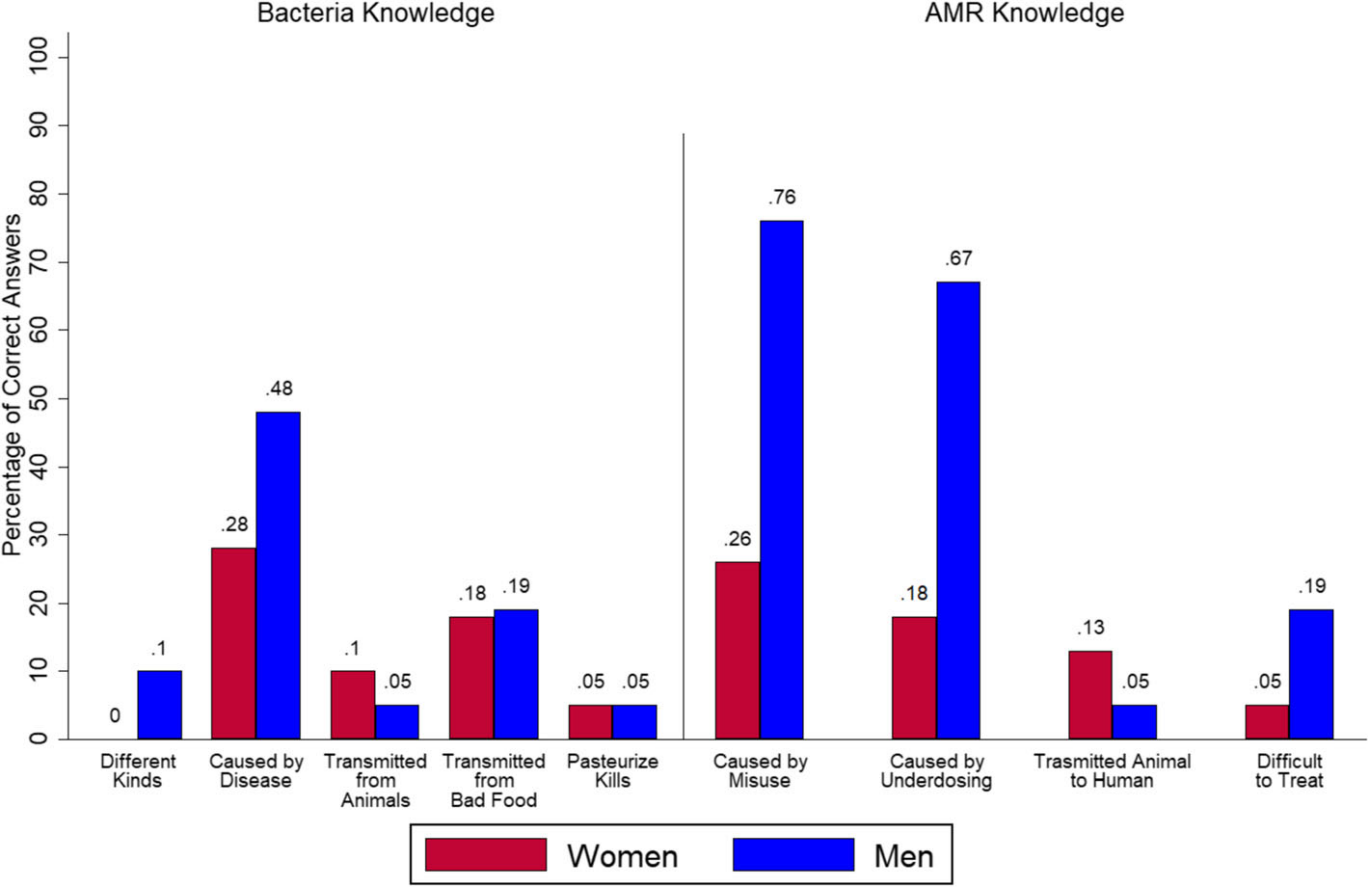
Correct Answers Assessing Knowledge of Bacteria and AMR by Gender. Proportion of participants providing correct responses when asked to provide information about bacteria and AMR during two-month follow-up evaluation

Assessment of knowledge retention demonstrated that men (*n* = 21) recalled significantly more bacteria and AMR knowledge items (0.30) than women (0.14) (*n* = 39, *P* = .03) (Fig. 1). Gender-based differences in AMR knowledge were due to the greater proportion of men reporting that underdosing contributes to emergence of resistance. More men also recalled that diseases caused by AMR organisms are difficult to treat.

Across both sexes, a one-unit increase in wealth and the number of AMR-specific knowledge items recalled were associated with rate ratio increases of recalled bacterial knowledge of 1.49 and 1.38, respectively, while holding other variables constant (see Additional file 3: Table S2). Among women, every additional child was associated with a rate ratio decrease by a factor of 0.707. Every additional year of age was associated with a rate ratio increase of 1.056 in bacteria knowledge recalled.

In terms of AMR-related knowledge, men, compared to women, recalled items at a 3.13 greater rate. Age was marginally significant (*P* = 0.06) in both men and women with the rate ratio of AMR knowledge recalled expected to decrease by a factor of 0.975. For men who used the innovation, the rate ratio of AMR recalled decreased by a factor of 0.355, although this effect was only marginally significant (*P* = 0.073) (see Additional file 3: Table S4).

**Fig. 2:**
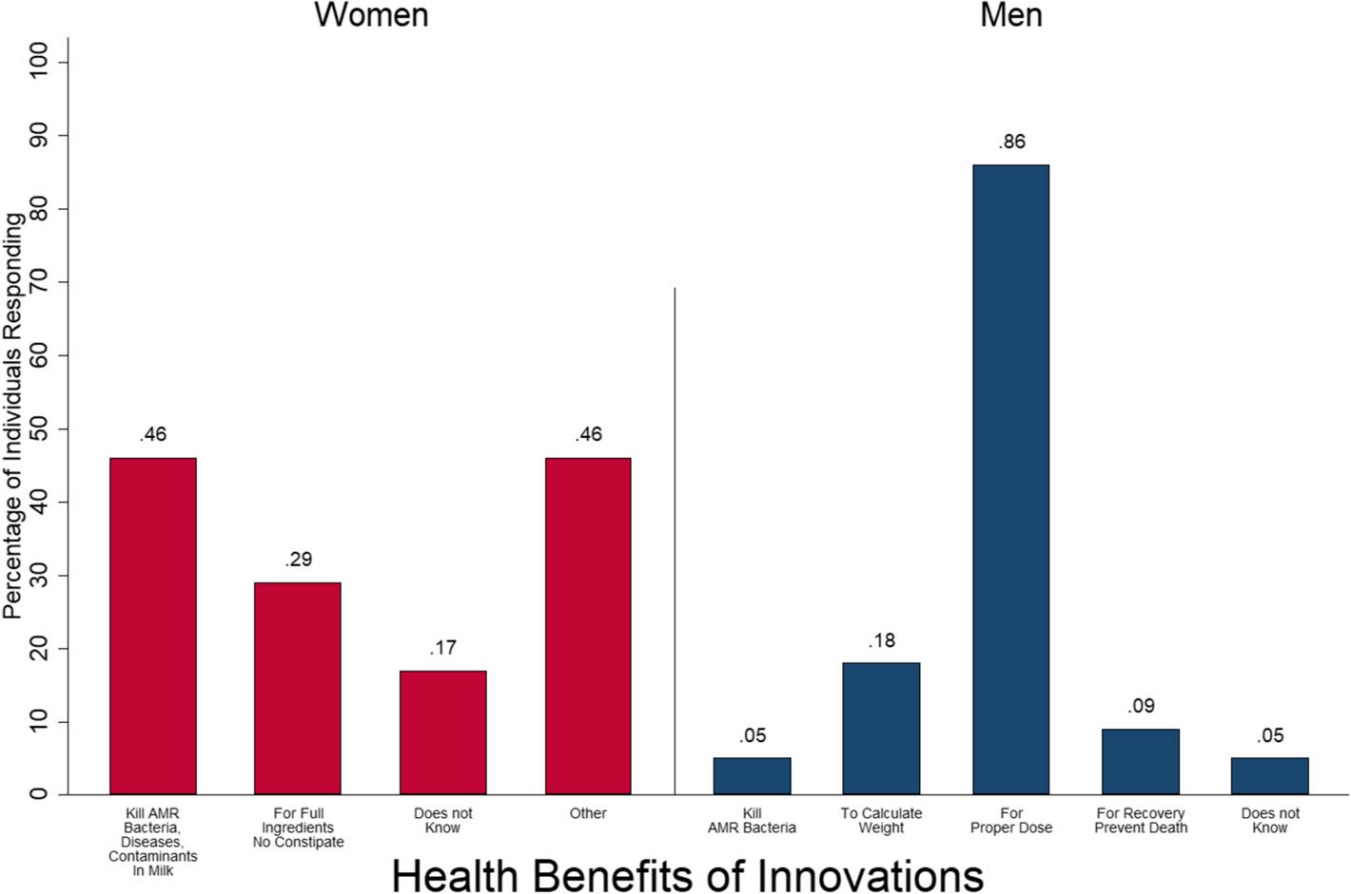
Individuals Reporting Health Benefits of Innovations. Proportion of individuals reporting each health benefit of the innovations. Women (red) were provided with thermometers for milk pasteurization. Men (blue) were provided with measuring tapes and dosing charts to calculate livestock weight to determine the proper dose of an antibiotic

Women reported one-third of correct answers for the health benefits of the thermometers while men reported a similar percentage (38%) of correct answers for the health benefits of the weight estimation materials (Fig. 2). Nobody mentioned all of the correct answers. Almost one-half of women mentioned other benefits not included in the core messages, including unspecified health benefit (0.20), it builds body or helps to gain weight (0.07), the milk tastes good (0.07), the milk increases body heat (0.02), the milk is “good” (0.10), and the milk increases energy (0.07). Several indicators were marginally related to recall. Women that have used the innovation were expected to have a rate of recall 1.764 times greater than those who did not use the innovation (*P* = 0.07). Every additional year of age was associated with a rate ratio increase of recall by a factor of 1.02 (*P* = 0.05). A unit increase in AMR Knowledge was associated with a rate ratio increase of recall by 1.26 (*P* = 0.05) (Additional file 3: Table S6.) A majority of men (86%) recalled that health benefits from using the weight estimation materials resulted from motivating proper dosage. Like women, men mentioned additional benefits including animal weight gain (0.05) and increasing the number of livestock (0.05). The number of correct responses was not significantly associated with any demographic or socioeconomic characteristics in men (Additional File 3: Table S6).

### Innovation use: Women and thermometers

Almost three-fourths of the women reported thermometer use. Each additional child increased the odds of using the thermometer over 5 times while older women were less likely to use the thermometers (See Additional file 3: Table S8). Of those that had used the thermometers, most had used them during the wet season (29%), which was immediately after we disseminated the thermometers. Two months later, during the drought season, only one woman was using a thermometer whereas most other women reported not using a thermometer because there was no milk (due to drought). Nevertheless, when there is milk available, 90% of women said that they pasteurize all of it except the portion meant for sour milk. Some women mentioned that they intend to pasteurize all milk, even milk for adults, when the cows return. A small percentage (10%) of women also mentioned pasteurizing milk for souring. Very few women reported difficulties using the thermometers and most reported that they like using the thermometers because pasteurized milk has a sweet or pleasant taste and it does not cause constipation or bloating like boiled milk.

In addition, the women in the pasteurization “pilot” study reported on their own additional experiments with the thermometers. They indicated that pasteurization tended to work best with larger quantities of milk (more than one liter). They reported that small quantities tended to be difficult to control as the milk went from pasteurization temperature to boiling very rapidly.

**Fig. 3:**
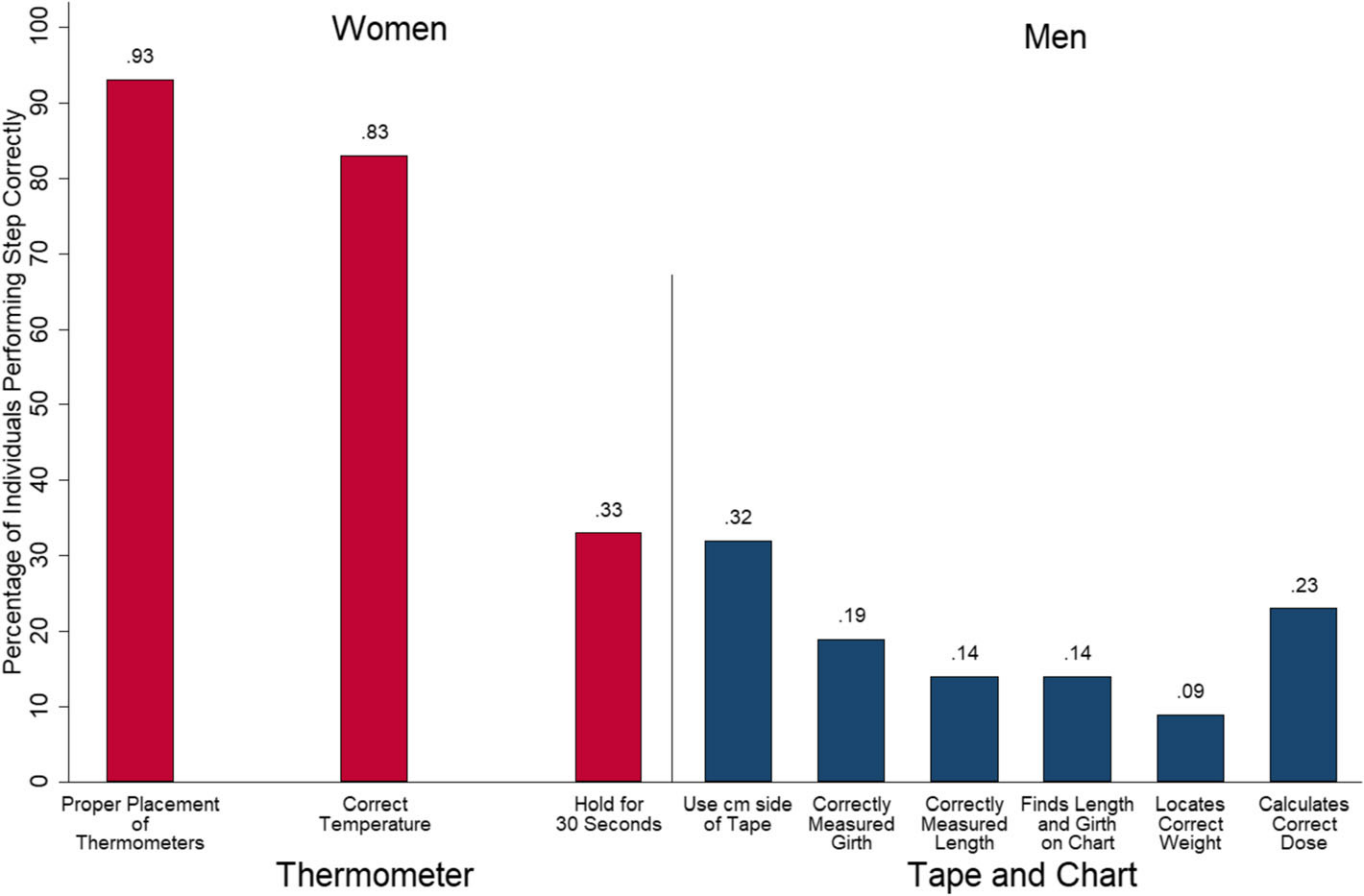
Correct steps for thermometers (women) and weight estimation materials (men). Proportion of participants performing each step of the innovation procedure correct. Women (red) were provided thermometers, which involved three steps. Men (blue) were provided with measuring tapes and dosing charts, which involved six steps

On average, women placed the thermometer correctly 93% of the time and noted the correct temperature 83% of time (Fig. 3). Only 33% remembered that it is necessary to wait 30 s once the temperature is reached for the pasteurization to complete. Poisson model results indicated that, for women, no demographic, socioeconomic, knowledge retention, or innovation use variables were associated with the number of correct steps performed.

### Innovation use: Men, measuring tapes, and charts

Over 40% of men used their charts and tapes, the majority of which (56%) indicated they used them every time they administered antibiotics (see Additional file 3: Table S5). Most participants (75%) mentioned that they like the innovation because it helps them to estimate an animal’s weight for proper treatment. Over 40% of participants, however, mentioned that the chart was difficult to read and/or understand. Men who had used their innovations did not significantly differ from those who had not used them except that those who used the innovations recalled significantly fewer AMR knowledge items.

On average, less than one-third of men performed the innovation steps correctly (Fig. 3). Men performed 18% of the tape and chart steps correctly and no steps were performed correctly more than 50% of the time (see Additional file 3: Table S4). Poisson model results indicated that, for men, education was positively associated with the number of correct steps performed with rate ratios for the number of correct steps performed expected to increase by a factor of 4.356 for every additional year of education (see Additional file 3: Table S8).

## Discussion

Overall, the Maasai were very eager to learn about and adopt the innovations. In terms of knowledge retention, most individuals could only recall one knowledge point about bacteria, antimicrobial resistance, and the associated health benefits of the innovations. Importantly, for future intervention efforts, retention of bacterial knowledge was positively associated with AMR knowledge, suggesting that AMR knowledge enforces retention of bacterial knowledge, which could prove beneficial for other health interventions (e.g., typhoid prevention). Overall, knowledge retention did not appear to motivate innovation use, with the exception that women who used their thermometers could recall significantly more health benefits of pasteurization. Among Maasai men, in contrast, the retention of bacteria knowledge most strongly predicted innovation use, albeit only marginally.

Around half of participants used their innovation, although few women were using the thermometers when we returned after two months. We suspect usage rates would be higher for both sets of innovations if we returned after the drought season had ended when more cattle are present to treat or to provide milk. Indeed, women stated their intention to pasteurize all milk, and over 70% performed all pasteurization steps correctly. In contrast, only 40% of men reported using the measuring tapes and dosage charts and only 18% performed the correct number of steps. Lower performance among men was partially due to men having more steps to perform than women and because the dosing chart was difficult to read and understand given 59% illiteracy among household heads [4]. These results suggest that intervention efforts promoting weight estimation may require more training among livestock-keepers with high illiteracy rates. In total, the varied retention of knowledge and skills emphasizes the importance of interventions that include reinforcement of information, possibly through channels such as radio and community health meetings.

Few demographic or socioeconomic variables were associated with knowledge retention or intervention use. Among women, however, use was related to having more children, suggesting a link between the motivation of maintaining child health, innovation use, and knowledge retention. These findings suggest that public health messages to limit AMR should stress the health benefits of similar innovations and how those benefits articulate with the beliefs and concerns of the study community. Indeed, culture is one factor among many (e.g. poor quality of antibiotics, misuse among physicians and lay people) that plays an important role in antibiotic use behavior [28]. For example, the belief that injections are more powerful than pills or that antibiotics can kill many diseases, beliefs that are Maasai share, appear to decrease health by increasing the likelihood of antibiotic misuse. Although we agree that part of the solution to limiting the emergence and spread of AMR in LMICs is unearthing the local beliefs and practices that are health lowering, it is also important to highlight local beliefs and practices that are health enhancing, or that can lead to health enhancing behaviors. Among Maasai, some of these values include local dietary preferences for unboiled milk, concerns over child health, and a desire to improve the health of livestock.

Among Maasai men, bacteria knowledge was positively correlated with wealth, indicating that wealthier men are more likely to retain bacteria knowledge (perhaps because they have more direct and/or indirect access to education), and therefore might be more motivated to use their innovations. Education was also strongly associated with the correct use of innovations, such that, in this population at least, lack of education remains a major obstacle to the prudent administration of antibiotics. Across LMICs, education appears to play an important role in the emergence and spread of AMR. In a meta-analysis of the burden, risk factors, and outcomes of antimicrobial use in developing countries, Ocan et al. [29] found that low education levels are consistently associated with antibiotic misuse (i.e. self-administration of antibiotics). Moreover, low education levels are often coupled with ineffective policies regulating over-the-counter access of antibiotics and a high burden of infectious diseases. All of these factors increase the likelihood of antibiotic misuse and the development of AMR. Our study further demonstrates that (low) education is a major obstacle for AMR interventions in LMICs. We hence performed public health messages and innovation demonstrations at the local primary school [J. Roulette et al. 2017, unpublished data]. Children, as key stakeholders who produce and reproduce community culture, are essential for sustaining health and disease-control efforts. Indeed, preliminary results of a Maasai network study on innovation sharing indicate that many men rely on their school-aged sons to help them use the charts and tapes [C. Roulette et al. 2017, unpublished data].

Finally, women in our “pilot” study reported results of their own experimentation that might also indicate another barrier to innovation use (i.e. thermometers) in poorer households. These women reported that pasteurization works best with more than 1 l of milk. Depending on the season, Maasai cows produce between 0.5 and 2 l(s) of milk for household consumption per day per lactating cow. Households with the smallest herds may therefore have insufficient volume of daily milk offtake for stable pasteurization, and may require different intervention strategies compared with wealthier households.

## Conclusion

Community-targeted interventions will be critical to decrease selection and maintenance of AMR in low-income regions such as SSA where top-down solutions (regulation of antibiotics, surveillance) are often untenable due to resource constraints. Public health models also increasingly recognize the need for community-level interventions that are collaborative and culturally-situated [8–11] . Community interventions, such as this project, are important bottom-up mechanisms for building social-ecological resilience to AMR [30]. Supported by recent studies finding the correlates of multidrug resistance vary within and across groups [31], we call for more localized, community- or culturally-specific interventions that increase the prudent use of antibiotics and increase health literacy as it pertains to pathogenic bacteria, use of antibiotics, and the development of AMR. Moreover, our results underscore the need to account for sociocultural and political-economic dynamics of local populations when designing community interventions. To effectively reduce selection for AMR in agro-pastoral communities, different health-innovation promotion strategies will be needed for men and women. For women, innovation presentations might target child health and dietary benefits of pasteurized versus boiled milk, while men would value livestock health and improved wealth more than women. Overall, the Maasai’s level of community participation with the project indicates their willingness to engage and reflects their enthusiasm for relevant public health interventions. While not everyone was using the innovations correctly, the Maasai nonetheless value the devices for their abilities to help them manage the health of their families and livestock.

The results of this study are complicated by the short duration between intervention and follow-up, which makes it difficult to draw firm conclusions regarding the long-term retention of knowledge and skills. Future research should further evaluate knowledge and skill retention and associated socioeconomic and demographic factors. Future interventions could also selectively target large families, in light of our finding that women with more children are more likely to have used the innovations. Moreover, there are several improvements that can be made to increase the usability of the innovations. For example, the weight estimation material might be packaged with additional instruction and demonstration, be simplified, or incorporated into an easy-to-use and highly accessible electronic format. New thermometers are already being developed and tested that simplify the pasteurization process and improve data collection. These improvements can open the door to larger scale interventions that could ultimately be linked to a measurable change in AMR rates.

## Additional files

Additional file 1: Community-Health Meeting Script, Innovation Use Protocols, and Pictures of the Innovations, Community-Meetings, and Training Sessions. Contains three sections. Includes three parts. Part 1 (Community-Health Meeting Script and Image) includes an example of the script from a community-health meeting and images from a meeting. Part 2 (Pasteurization Protocol and Images) includes protocols (in English) for using the thermometers for pasteurization as well as pictures of a pasteurization training session. Part 3 (Weight-estimation Protocol and Images of Materials and Training) includes protocols for using the weight-estimation material (in Swahili), an image of the dosing chart, and pictures of a weight-estimation training session. (DOCX 874 kb)

Additional file 2: A Table of the Community-Meeting Script and its Correspondence to the Follow-Up Evaluation Questions and a Table of the Follow-up Evaluation Questionnaire. Includes two tables. Table S1 consists of the two of the knowledge domains (bacteria and AMR) and the associated knowledge items. Each knowledge item is shown next to its corresponding script from the community-health meetings. Table S2 is the follow-up evaluation questionnaire and consists of 26 questions (in English). (DOCX 18 kb)

Additional file 3: Full Poisson and Logit Models, with Model fit Indices and Variance Inflation Factors (VIFs) for all Models Full models include: Poisson Models of the number of bacteria (Table S1) and AMR (Table S2) knowledge items recalled versus demographic and socioeconomic variables, for all participants combined, women, and men; Poisson models of the number of health benefit knowledge items recalled (Table S3) and the number of innovation use steps performed correctly (Table S4) versus demographic and socioeconomic variables, by gender; and a logit model of whether an innovation was used (1) or not (0) versus demographic and socioeconomic variables, by gender (Table S5). (DOCX 307 kb)

## Abbreviations

AMR: Antimicrobial Resistance
*E. coli*: *Escherichia coli*
EVK: Ethnoveterinary knowledge
LMIC: Low- and middle-income countries
NSF: National Science Foundation
OTC: Oxytetracycline
SSA: Sub-Saharan Africa
TBC: Total bacterial count
VIF: Variance inflation factor

## Footnotes

1 We used 30 s as a conservative recommendation given heat-treatment is an elevation-dependent process (i.e., milk will boil at a lower temperature at higher elevations).

2 We were only able to obtain measuring tapes with centimeters on one side and inches on the reverse side.
