# Supplementary material for: A two-month follow-up evaluation testing interventions to limit the emergence and spread of antimicrobial resistant bacteria among Maasai of northern Tanzania
Source: BMC Infect Dis. 2017 Dec 15;17:770. doi: 10.1186/s12879-017-2857-z (PMC5732506; doi:10.1186/s12879-017-2857-z)

**Additional File 1**

**TITLE: Community-Health Meeting Script, Innovation Use Protocols, and Pictures of the Innovations**

**Public-Health Meeting Script**

Thank you all, etc. Make sure everyone is introduced etc.

We are working on a research project about bacteria in the Maasai and their animals. Bacteria are everywhere. Not all bacteria are bad. For example, there are bacteria in our gut that help digest food. However, some bacteria can be harmful, especially for young children.

We collected fecal samples to test for bacteria that are “resistant” to OTC and other medicines.

This bacteria is called E. coli, and usually it is not harmful, but it acts like other bacteria that are harmful.

Sometimes when a person or animal takes a medicine for a long time or if the medicine is not given the right way, then the animal can get used to the medicine and it will not work very well.

The same kind of thing is very common in bacteria. Mostly the medicine kills the bacteria, but bacteria can get used to or “resistant” to the medicine and the medicine will not work well.

This can happen if you use the medicine the wrong way, like give doses that are too small or if you don’t use it long enough. Then the bacteria will become very hard to kill and the animal may not get better.

Bacteria from a cow or other animal can move to humans too. If you touch the feces bacteria can get on your hand and into your stomach. If that bacteria is “resistant” or used to OTC or another medicine then people can get sick.

We have tested to see if Maasai people and animals have bacteria that are resistant to medicines like OTC.

This is a very big question, not just for the Maasai but for all people. It is a big question for science and this study may help millions of people around the world.

Two results we have so far and that I want to share with you today are:

1. There are more resistant bacteria in Maasai than in Chaga and Warusha. We are trying to find out all of the reasons why this is the case, but right now we think it is because of milk.

There are a lot of bacteria in Maasai milk. We have found that when Maasai people drink a lot of milk that is not boiled, then the people get resistant bacteria too. Maasai people say they don’t like the test of boiled milk and it does not clot well. We have found a way to treat milk (the same way we treat milk in America) that kills the bacteria without boiling. It is called “Pasteurization” and we will show you how to do it with your milk at home. All you have to do is heat the milk to 75 degrees c for 30 seconds. One of the main goals of today is to show you how to pasteurize your milk.

1. It is important that that you do not use the milk or urine of a cow that has been treated with OTC within the last week. If you must use the milk immediately, even if you are using it to feed dogs, you should boil it for ten minutes then let it cool. You can test how cool the milk is with your finger, but make sure to rinse your finger with water first.

If you have any questions at this time please feel free to ask. We might not have all of the answers to your questions today, but we will write down your questions and ask the appropriate researchers after we leave today. When we get the answers we will contact you and provide you with the information. This might take a week or so.

**Part 2: Pasteurization Protocol and Images**

**Pasteurization Protocol (in English):**

To pasteurize your milk all you have to do is heat the milk to 75 degrees c for 30 seconds. It is best if you use more than a liter of milk. If you use less the milk can become very watery and it will not clot well. It is also important that you watch the temperature very carefully. If it gets too hot then it is like boiling it and it will not clot well.

If the pot gets too hot you can remove it from the heat for a few seconds, or use less firewood. You must heat the milk for 30 seconds so if you remove the pot from the fire during pasteurization make sure the temperature remains at or above 75 degrees.

We will demonstrate how to pasteurize the milk. We will also pass out thermometers today. These thermometers are yours to keep and to share with your family and friends. The thermometers are also numbered. We would like to write down your name when we hand you a thermometer. The purpose of numbering the thermometers and writing down your name is so that when we return in a few months we can learn how Maasai use and share the thermometers. This information will be used to help us develop more effective health projects for the Maasai community in the future.

**Figure 2. Pictures of thermometer**

**
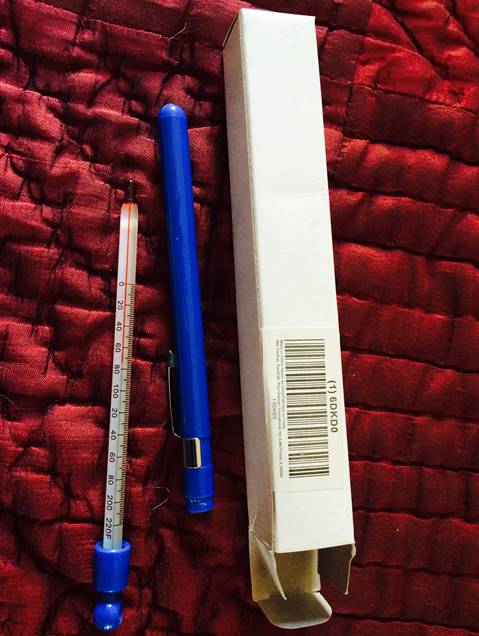
**

**Part 3: Weight-estimation protocol and images of materials**

**Figure 3. Weight-estimation materials protocol (Swahili translated version)**

**Figure 4. Dosing chart**


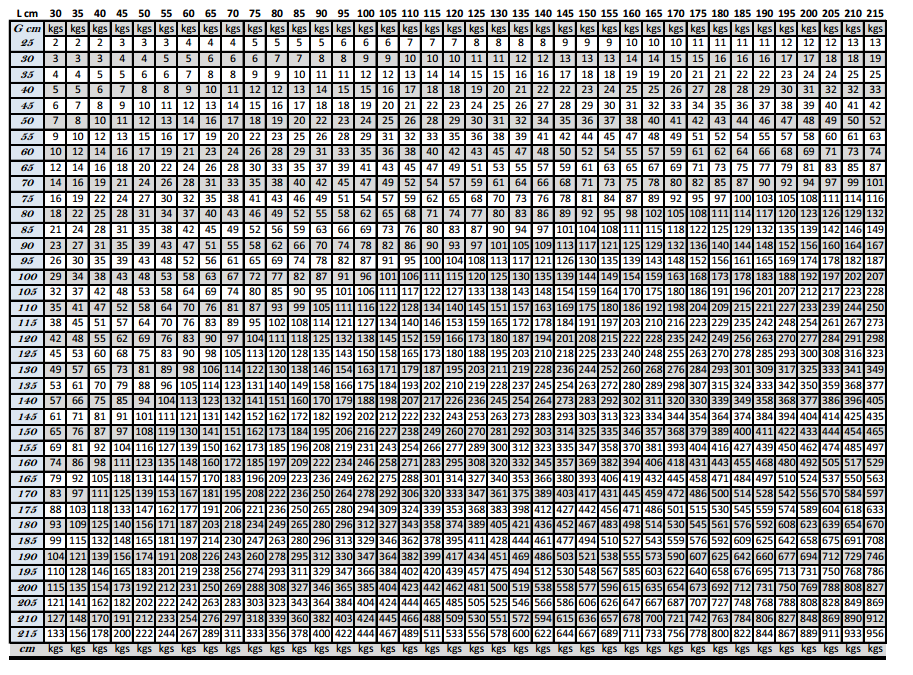

Supplement: Supplementary file 1 — Community-Health Meeting Script, Innovation Use Protocols, and Pictures of the Innovations, Community-Meetings, and Training Sessions. Contains three sections. Includes three parts. Part 1 (Community-Health Meeting Script and Image) includes an example of the script from a community-health meeting and images from a meeting. Part 2 (Pasteurization Protocol and Images) includes protocols (in English) for using the thermometers for pasteurization as well as pictures of a pasteurization training session. Part 3 (Weight-estimation Protocol and Images of Materials and Training) includes protocols for using the weight-estimation material (in Swahili), an image of the dosing chart, and pictures of a weight-estimation training session. (DOCX 874 kb) [file 12879_2017_2857_MOESM1_ESM.docx]
