# Supplementary material for: A two-month follow-up evaluation testing interventions to limit the emergence and spread of antimicrobial resistant bacteria among Maasai of northern Tanzania
Source: BMC Infect Dis. 2017 Dec 15;17:770. doi: 10.1186/s12879-017-2857-z (PMC5732506; doi:10.1186/s12879-017-2857-z)
Supplement: Supplementary file 2 — A Table of the Community-Meeting Script and its Correspondence to the Follow-Up Evaluation Questions and a Table of the Follow-up Evaluation Questionnaire. Includes two tables. Table S1 consists of the two of the knowledge domains (bacteria and AMR) and the associated knowledge items. Each knowledge item is shown next to its corresponding script from the community-health meetings. Table S2 is the follow-up evaluation questionnaire and consists of 26 questions (in English). (DOCX 18 kb) [file 12879_2017_2857_MOESM2_ESM.docx]

**Additional File 2**

**Title:** **A Table of the Community-Meeting Script and its Correspondence to the Follow-Up Evaluation Questions and a Table of the Follow-up Evaluation Questionnaire**

**Table S1. Dissemination material correspondence to follow-up evaluation questions**

|  | Script from dissemination material |
| --- | --- |
| Bacteria |  |
| (i) Variety of kinds | -“Bacteria are everywhere. Not all bacteria are bad. For example, there are bacteria in our gut that help digest food.” |
| (ii) They cause disease | -“Some bacteria can be harmful, especially for young children” |
| (iII) They can be transmitted | -“Bacteria from a cow or other animal can move to humans too. If you touch the feces bacteria can get on your hand and into your stomach. If that bacteria is “resistant” or used to OTC or another medicine then people can get sick”…  -“We have found that when Maasai people drink a lot of milk that is not boiled, then the people get resistant bacteria too.” |
| (Iv) Pasteurization/boiling kills bacteria in milk | -We have found a way to treat milk (the same way we treat milk in America) that kills the bacteria without boiling. It is called “Pasteurization” |
| Antibiotic Resistance |  |
| (i) Caused by improper use of antibiotics | -“Sometimes when a person or animal takes a medicine for a long time or if the medicine is not given the right way, then the animal can get used to the medicine and it will not work very well. The same kind of thing is very common in bacteria. Mostly the medicine kills the bacteria, but bacteria can get used to or “resistant” to the medicine and the medicine will not work well.”  -“This can happen if you use the medicine the wrong way, like give doses that are too small or if you don’t use it long enough”  -“It is important that that you do not use the milk or urine of a cow that has been treated with OTC within the last week.” |
| (ii) Can be transmitted from animal to human | -“Bacteria from a cow or other animal can move to humans too. If you touch the feces bacteria can get on your hand and into your stomach. If that bacteria is “resistant” or used to OTC or another medicine then people can get sick.“ |
| (iii) Diseases resistant to medicines and difficult to treat | -“Then the bacteria will become very hard to kill and the animal may not get better.” |

**Table S2. Follow-up evaluation questionnaire**

|  | **No.** | **Female** | **Male** |
| --- | --- | --- | --- |
| **Controls** | **1** | Age | Age |
|  | **2** | Gender | Gender |
|  | **3** | How many years of school have you completed? | How many years of school have you completed? |
|  | **4** | Are you married? | Are you married? |
|  | **5** | How many cowives do you have? | How many wives do you have? |
|  | 6 | Do you have children? | Do you have children? |
|  | 7 | How many children do you have? | How many children do you have? |
|  | 8 | How many heads of cattle are owned by the boma? | How many heads of cattle are owned by the boma? |
|  | 9 | Is there a pit toilet in boma? | Is there a pit toilet in boma? |
|  | 10 | Is there a cinder block house in boma? | Is there a cinder block house in boma? |
| **Innovation adoption and use** | **11** | Do you use the thermometer? | Do you use the chart and measuring tapes? |
|  | **12** | Do you use it every time you prepare milk to drink? | Do you use them every time you give antibiotics to livestock? |
|  | **13** | When you prepare drinking milk, when are you most likely to use the thermometer? | When you give antibiotic meds to livestock, when are you most likely to use chart and tapes? |
|  | **14** | When you prepare drinking milk, when are you least likely to use the thermometer? | When you give antibiotic meds to livestock, when are you least likely to use chart and tapes? |
|  | **15** | In your opinion, what are some of the problems of using the thermometer? | In your opinion, what are some of the problems of using the chart and tapes? |
|  | **16** | In your opinion, what are some of the benefits of using the thermometer? | In your opinion, what are some of the benefits of using the chart and tapes? |
| **Retention of Knowledge** | **17** | Can you show me or explain to me how you use the thermometer? (three steps total) | Can you show me or explain to me how you use the chart and tapes? (six steps total) |
|  | **18** | Why did we provide the thermometer to you? | Why did we provide the chart and tapes to you? |
|  | **19** | What health problems might it help solve? | What health problems might it help solve? |
|  | **20** | What are bacteria? | What are bacteria? |
|  | **21** | What is antibiotic resistance? | What is antibiotic resistance? |
| **Transmission of knowledge and innovation** | **22** | Do (or did) you share your thermometer with anyone? | Do (or did) you share your chart and tapes with anyone? |
|  | 23 | Can you provide their name and tell us where they live? | Can you provide their name and tell us where they live? |
|  | **24** | How they are related to you? | How they are related to you? |
|  | **25** | How often, or when, do they borrow usually borrow the innovation? | How often, or when, do they borrow usually borrow the innovation? |
|  | **26** | Did you explain to them how to use it? | Did you explain to them how to use it? |
