# Supplementary material for: A two-month follow-up evaluation testing interventions to limit the emergence and spread of antimicrobial resistant bacteria among Maasai of northern Tanzania
Source: BMC Infect Dis. 2017 Dec 15;17:770. doi: 10.1186/s12879-017-2857-z (PMC5732506; doi:10.1186/s12879-017-2857-z)

**Additional File 3.**

**Title: Full Poisson and Logit Models, with Model fit Indices and Variance Inflation Factors (VIFs) for all Models**

**Appendix B. Analysis and Model Fit.**

Generalized linear models were used to examine the demographic and socioeconomic variables associated with knowledge, correct steps performed, and innovation use. Knowledge items were specified using Poisson models as these variables are counts and their distribution displayed positive skew but did not show evidence of significant overdispersion. Coefficients in Poisson models are incidence rate ratios, here, the rate at which additional knowledge items or correct steps are recalled/performed. Logistic regression was used to determine characteristics associated with innovation use. Coefficients of logit models are presented as odds ratios where ratios above 1 indicate a greater odds of innovation use and below 1 indicating lower odds of innovation use.

Models are first presented as full models and then reduced stepwise models. Model diagnostics include multicollinearity and comparative and global indices of model fit. Multicollinearity was assessed using variance inflation factors with the package *collin*. Stata 13 (StataCorp 2012) was used for all analyses.

**Table S1. Knowledge of Bacteria in Both Genders, Women, and Men**. Full Model with all covariates included.

|  | Both Genders |  | Women |  | Men |  |
| --- | --- | --- | --- | --- | --- | --- |
|  | B | SE | B | SE | B | SE |
| Age | 1.021 | 0.991 - 1.051 | 1.062 | 1.008 - 1.118* | 1.016 | 0.955 - 1.080 |
| Sex (0=female, 1=male) | 1.294 | 0.632 - 2.648 |  |  |  |  |
| Education (0=no, 1=yes) | 0.928 | 0.500 - 1.722 | 0.931 | 0.390 - 2.223 | 1.334 | 0.485 - 3.667 |
| Num Wives or Co-Wives | 0.972 | 0.803 - 1.178 | 0.933 | 0.764 - 1.141 | 1.118 | 0.553 - 2.260 |
| Total children | 0.933 | 0.857 - 1.015 | 0.713 | 0.557 - 0.913** | 0.951 | 0.821 - 1.101 |
| Total cattle | 1.002 | 0.999 - 1.005 | 1.005 | 1.000 - 1.010 | 1.000 | 0.995 - 1.005 |
| Wealth | 1.511 | 1.122 - 2.033** | 1.335 | 0.892 - 1.998 | 1.730 | 0.988 - 3.030 |
| Used Innov. (0=no, 1=yes) | 1.910 | 0.961 - 3.796 | 2.472 | 0.875 - 6.979 | 2.029 | 0.596 - 6.905 |
| AMR Knowledge | 1.377 | 1.015 - 1.868* | 1.256 | 0.839 - 1.880 | 1.500 | 0.822 - 2.738 |
| Pseudo R2 | 0.08 |  | 0.13 |  | 0.11 |  |
| Prob>chi2 | 0.17 |  | 0.08 |  | 0.56 |  |
| Log Likelihood | -78.07 |  | -46.39 |  | -27.37 |  |
| LR chi2 | 12.90 |  | 13.89 |  | 6.76 |  |
| Obs | 62 |  | 40 |  | 22 |  |

* *p*<0.05; ** *p*<0.01

**Table S2. Knowledge of Bacteria in Both Genders, Women, and Men**. Reduced models using stepwise backward regression (0.20 retention factor).

|  | Both Genders |  | Women |  | Men |  |
| --- | --- | --- | --- | --- | --- | --- |
|  | B | SE | B | SE | B | SE |
| Age | 1.018 | 0.992 - 1.045 | 1.056 | 1.006 - 1.108* |  |  |
| Sex (0=female, 1=male) |  |  |  |  |  |  |
| Education (0=no, 1=yes) |  |  |  |  |  |  |
| Num Wives or Co-Wives |  |  |  |  |  |  |
| Total children | 0.955 | 0.889 - 1.024 | 0.707 | 0.555 - 0.899** |  |  |
| Total cattle |  |  | 1.004 | 1.000 - 1.009 |  |  |
| Wealth | 1.493 | 1.118 - 1.995** | 1.307 | 0.883 - 1.937 | 1.615 | 1.040 - 2.508* |
| Used Innov. (0=no, 1=yes) | 1.664 | 0.871 - 3.178 | 2.438 | 0.916 - 6.484 |  |  |
| AMR Knowledge | 1.387 | 1.058 - 1.819* |  |  |  |  |
| Pseudo R2 | 0.06 |  | 0.12 |  | 0.06 |  |
| Prob>chi2 | 0.05 |  | 0.03 |  | 0.05 |  |
| Log Likelihood | -79.06 |  | -47.07 |  | -28.80 |  |
| LR chi2 | 10.93 |  | 12.54 |  | 3.90 |  |
| Obs | 62 |  | 40 |  | 22 |  |

* *p*<0.05; ** *p*<0.01

**Figure 1. Fit Statistics Comparing Full and Reduced Models for Knowledge of Bacteria in Both Genders.** Reduced model indices are in “Current” column and Full in “Saved” column.


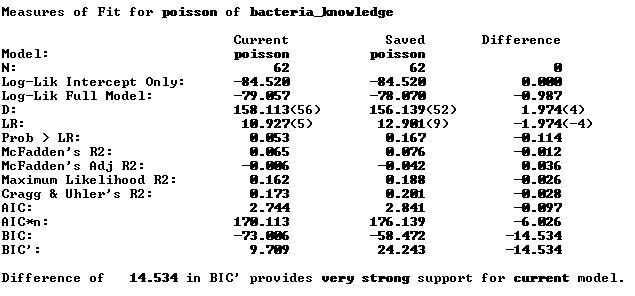


**Figure 2. Collinearity Measures for Knowledge of Bacteria in Both Genders.**


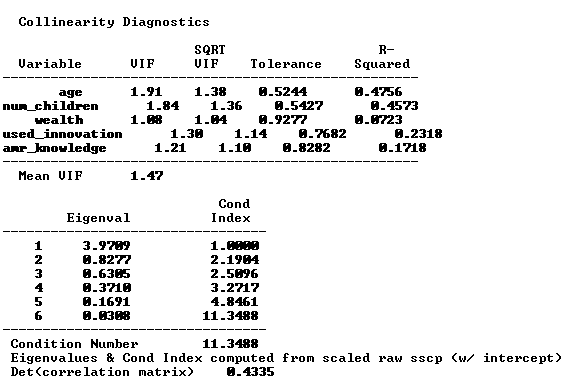


**Figure 3. Fit Statistics Comparing Full and Reduced Models for Knowledge of Bacteria in Women.** Reduced model indices are in “Current” column and Full in “Saved” column.


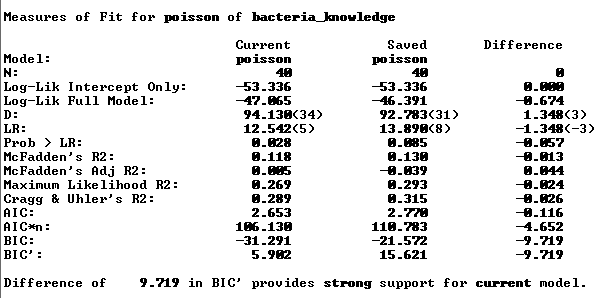


**Figure 4. Collinearity Measures for Knowledge of Bacteria in Women.**


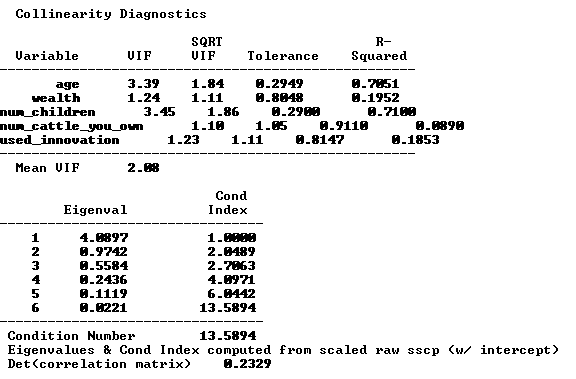


**Figure 5. Fit Statistics Comparing Full and Reduced Models for Knowledge of Bacteria in Men.** Reduced model indices are in “Current” column and Full in “Saved” column.


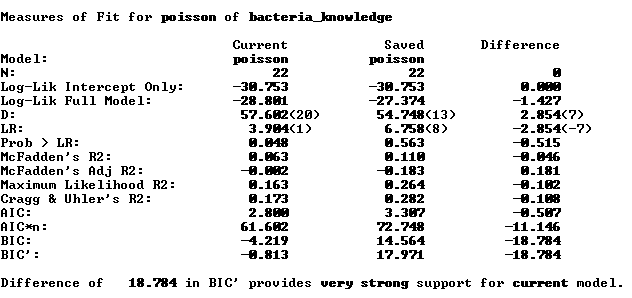


Note: No VIF information for men given only one variable was retained.

**Table S3. Knowledge of Antimicrobial Resistance in Both Genders, Women, and Men.** Full Model with all covariates included.

|  | Both Genders |  | Women |  | Men |  |
| --- | --- | --- | --- | --- | --- | --- |
|  | B | SE | B | SE | B | SE |
| Age | 0.963 | 0.929 - 0.999* | 0.972 | 0.911 - 1.038 | 0.956 | 0.901 - 1.014 |
| Sex (0=female, 1=male) | 2.719 | 1.340 - 5.520** |  |  |  |  |
| Education (0=no, 1=yes) | 1.041 | 0.582 - 1.862 | 1.123 | 0.449 - 2.808 | 1.144 | 0.489 - 2.674 |
| Num Wives or Co-Wives | 1.149 | 0.947 - 1.393 | 1.247 | 0.977 - 1.590 | 1.062 | 0.621 - 1.815 |
| Total children | 1.037 | 0.961 - 1.119 | 0.954 | 0.722 - 1.261 | 1.062 | 0.943 - 1.196 |
| Total cattle | 0.999 | 0.996 - 1.002 | 0.991 | 0.975 - 1.008 | 0.999 | 0.996 - 1.002 |
| Wealth | 0.772 | 0.515 - 1.158 | 0.782 | 0.447 - 1.368 | 0.920 | 0.461 - 1.838 |
| Used Innov. (0=no, 1=yes) | 0.500 | 0.259 - 0.966* | 0.832 | 0.280 - 2.471 | 0.355 | 0.135 - 0.933* |
| Bacteria Knowledge | 1.218 | 0.962 - 1.541 | 1.183 | 0.831 - 1.685 | 1.162 | 0.811 - 1.665 |
| Pseudo R2 | 0.15 |  | 0.08 |  | 0.12 |  |
| Prob>chi2 | 0.00 |  | 0.53 |  | 0.47 |  |
| Log Likelihood | -70.44 |  | -40.06 |  | -28.63 |  |
| LR chi2 | 25.03 |  | 7.08 |  | 7.62 |  |
| Obs | 62 |  | 40 |  | 22 |  |

**Table S4. Knowledge of Antimicrobial Resistance in Both Genders, Women, and Men**. Reduced models using stepwise backward regression (0.20 retention factor).

|  | Both Genders |  | Women |  | Men |  |
| --- | --- | --- | --- | --- | --- | --- |
|  | B | SE | B | SE | B | SE |
| Age | 0.975 | 0.950 - 1.001 | 0.970 | 0.931 - 1.009 |  |  |
| Sex (0=female, 1=male) | 3.131 | 1.632 - 6.008** |  |  |  |  |
| Used Innov. (0=no, 1=yes) | 0.635 | 0.369 - 1.095 |  |  | 0.500 | 0.234 - 1.067 |
| Num Wives or Co-Wives | 1.136 | 0.952 - 1.355 | 1.186 | 0.953 - 1.476 |  |  |
| Pseudo R2 | 0.13 |  | 0.04 |  | 0.05 |  |
| Prob>chi2 | 0.00 |  | 0.19 |  | 0.06 |  |
| Log Likelihood | -72.32 |  | -41.95 |  | -30.67 |  |
| LR chi2 | 21.27 |  | 3.29 |  | 3.54 |  |
| Obs | 62 |  | 40 |  | 22 |  |

**Figure 6. Fit Statistics Comparing Full and Reduced Models for Knowledge of Antimicrobial Resistance in Both Genders.** Reduced model indices are in “Current” column and Full in “Saved” column.


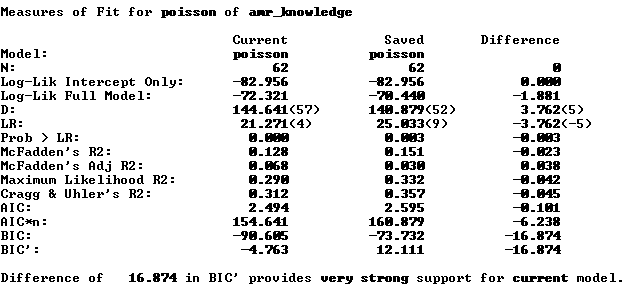


**Figure 7. Collinearity Statistics for Knowledge of Antimicrobial Resistance in Both Genders.**


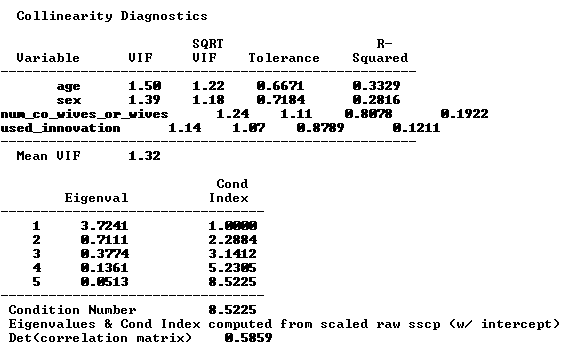


**Figure 8. Fit Statistics Comparing Full and Reduced Models of Knowledge of Antimicrobial Resistance for Women.** Reduced model indices are in “Current” column and Full in “Saved” column.


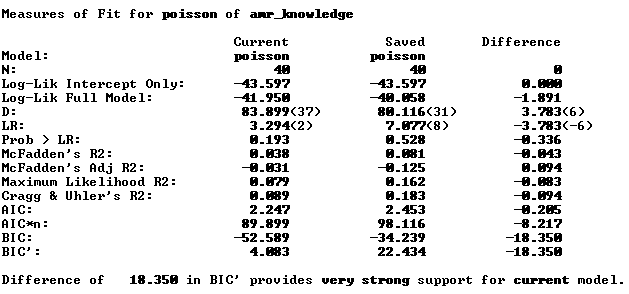


**Figure 9. Collinearity Statistics for Knowledge of Antimicrobial Resistance in Women.**


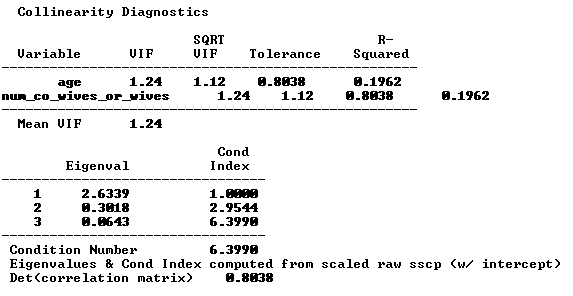


**Figure 10. Fit Statistics Comparing Full and Reduced Models for Knowledge of Antimicrobial Resistance in Men.** Reduced model indices are in “Current” column and Full in “Saved” column.


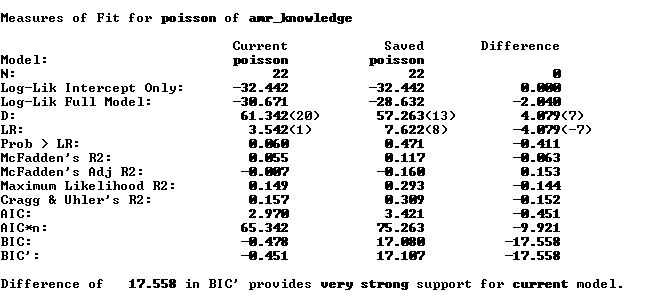


**Table S5. Number of Recalled Health Benefits of Innovations in Women and Men.** Full model with all covariates included.

|  | Women |  | Men |  |
| --- | --- | --- | --- | --- |
|  | B | CI | B | CI |
| Age | 1.039 | 1.003 - 1.077* | 1.021 | 0.980 - 1.063 |
| Education (0=no, 1=yes) | 1.096 | 0.631 - 1.904 | 1.234 | 0.554 - 2.748 |
| Num Wives or Co-Wives | 0.967 | 0.833 - 1.122 | 0.836 | 0.457 - 1.531 |
| Total children | 0.899 | 0.764 - 1.057 | 0.961 | 0.852 - 1.083 |
| Total cattle | 1.001 | 0.996 - 1.006 | 1.002 | 0.999 - 1.005 |
| Wealth | 1.053 | 0.758 - 1.463 | 1.212 | 0.677 - 2.170 |
| Used Innov. (0=no, 1=yes) | 2.164 | 1.008 - 4.646* | 1.577 | 0.580 - 4.284 |
| Bacteria Knowledge | 0.977 | 0.773 - 1.235 | 1.083 | 0.771 - 1.521 |
| AMR Knowledge | 1.278 | 0.972 - 1.680 | 1.203 | 0.731 - 1.981 |
| Pseudo R2 | 0.08 |  | 0.07 |  |
| Prob>chi2 | 0.33 |  | 0.90 |  |
| Log Likelihood | -55.35 |  | -28.29 |  |
| LR chi2 | 10.24 |  | 4.15 |  |
| Obs | 40 |  | 22 |  |

**Table S6. Number of Recalled Health Benefits of Innovations in Women and Men**. Reduced models using stepwise backward regression (0.20 retention factor).

|  | Women |  | Men |  |
| --- | --- | --- | --- | --- |
|  | B | CI | B | CI |
| Age | 1.017 | 1.000 - 1.034 |  |  |
| Used Innov. (0=no, 1=yes) | 1.745 | 0.913 - 3.337 |  |  |
| AMR Knowledge | 1.262 | 0.978 - 1.630 |  |  |
| Pseudo R2 | 0.07 |  |  |  |
| Prob>chi2 | 0.04 |  |  |  |
| Log Likelihood | -56.31 |  | -38.36 |  |
| LR chi2 | 8.32 |  | 0.0 |  |
| Obs | 40 |  | 22 |  |

**Figure 11. Fit Statistics Comparing Full and Reduced Models for Number of Recalled Health Benefits of Innovation in Women.** Reduced model indices are in “Current” column and Full in “Saved” column.


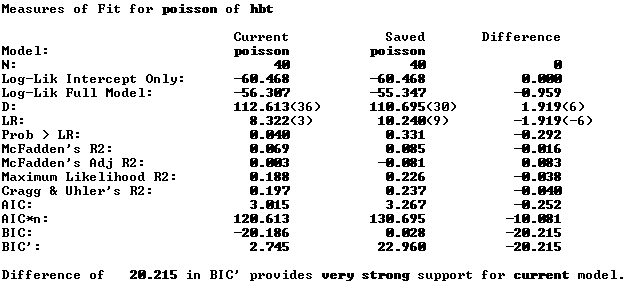


**Figure 12. Collinearity Statistics for Number of Recalled Health Benefits of Innovation in Women.**


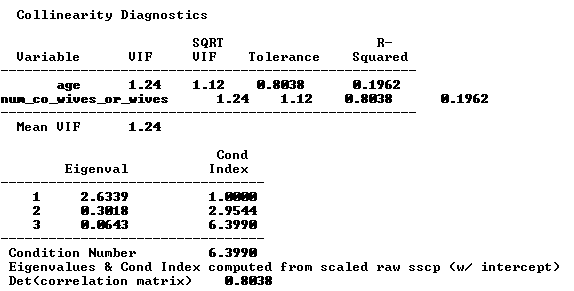


**Figure 13. Fit Statistics Comparing Full and Reduced Models for Number of Recalled Health Benefits of Innovation in Men.** Reduced model indices are in “Current” column and Full in “Saved” column.


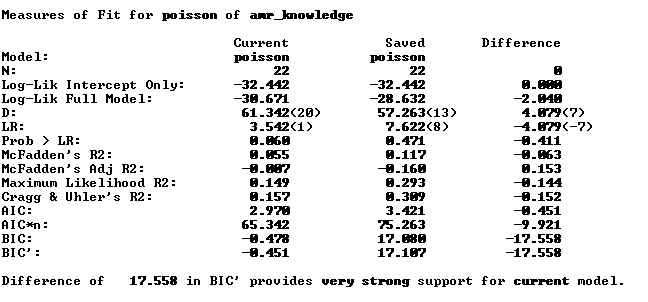


Note: No VIF information for men given no variable was retained.

**Table 7S. Number of Steps Performed Correctly for Innovations in Women and Men.** Full model with all covariates included.

|  | Women |  | Men |  |
| --- | --- | --- | --- | --- |
|  | B | CI | B | CI |
| Age | 0.993 | 0.961 - 1.027 | 0.994 | 0.934 - 1.057 |
| Education (0=no, 1=yes) | 0.890 | 0.543 - 1.459 | 4.148 | 1.299 - 13.248* |
| Num Wives or Co-Wives | 1.026 | 0.887 - 1.188 | 1.221 | 0.590 - 2.524 |
| Total children | 1.049 | 0.904 - 1.218 | 0.965 | 0.804 - 1.158 |
| Total cattle | 0.994 | 0.987 - 1.001 | 0.999 | 0.993 - 1.006 |
| Wealth | 1.021 | 0.772 - 1.350 | 1.577 | 0.775 - 3.211 |
| Used Innov. (0=no, 1=yes) | 0.998 | 0.549 - 1.814 | 0.916 | 0.228 - 3.678 |
| Bacteria Knowledge | 1.096 | 0.888 - 1.354 | 1.326 | 0.868 - 2.024 |
| AMR Knowledge | 0.998 | 0.777 - 1.282 | 0.858 | 0.436 - 1.690 |
| Pseudo R2 | 0.04 |  | 0.23 |  |
| Prob>chi2 | 0.85 |  | 0.11 |  |
| Log Likelihood | -55.34 |  | -23.60 |  |
| LR chi2 | 4.79 |  | 14.30 |  |
| Obs | 40 |  | 22 |  |
| Obs | 40 |  | 22 |  |

**Table 8S. Number of Steps Performed Correctly for Innovations in Women and Men**. Reduced models using stepwise backward regression (0.20 retention factor).

|  | Women |  | Men |  |
| --- | --- | --- | --- | --- |
|  | B | CI | B | CI |
| Total cattle | 0.995 | 0.989 - 1.001 |  |  |
| Wealth |  |  | 1.638 | 0.816 - 3.287 |
| Education (0=no, 1=yes) |  |  | 4.356 | 1.552 - 12.228** |
| Bacteria Knowledge |  |  | 1.271 | 0.882 - 1.832 |
| Pseudo R2 | 0.03 |  | 0.21 |  |
| Prob>chi2 | 0.07 |  | 0.00 |  |
| Log Likelihood | -56.07 |  | -24.16 |  |
| LR chi2 | 3.32 |  | 13.19 |  |
| Obs | 40 |  | 22 |  |

**Figure 14. Fit Statistics Comparing Full and Reduced Models for Number of Steps Performed Correctly in Women.** Reduced model indices are in “Current” column and Full in “Saved” column.


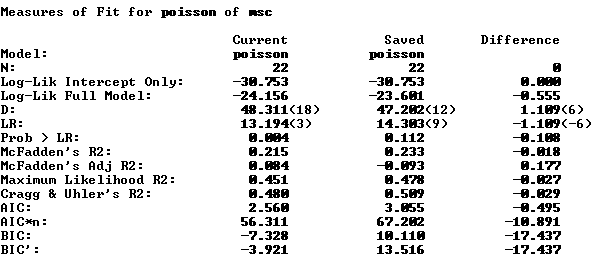


**Figure 15. Collinearity Statistics for Number of Steps Performed Correctly in Women.**


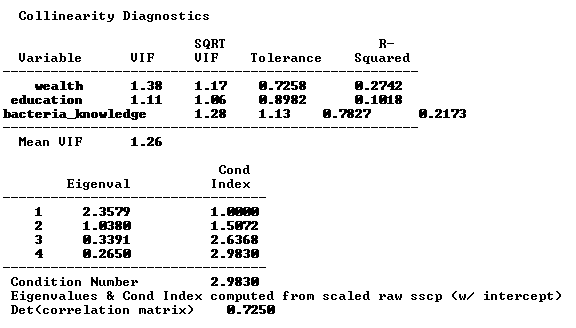


**Figure 16. Fit Statistics Comparing Full and Reduced Models for Number of Steps Performed Correctly in Men.** Reduced model indices are in “Current” column and Full in “Saved” column.


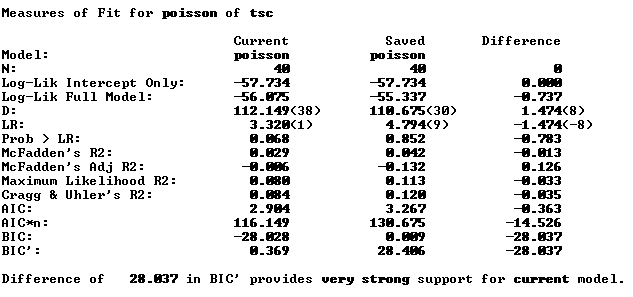


Note: No VIF information for men given no variable was retained.

**Table S9. Correlates of Innovation Use in Women and Men.** Full model with all covariates included.

|  | Women |  | Men |  |
| --- | --- | --- | --- | --- |
|  | B | SE | B | SE |
| Age | 0.790 | 0.627 - 0.996* | 0.554 | 0.236 - 1.301 |
| Education (0=no, 1=yes) | 0.368 | 0.044 - 3.076 | 7.064 | 0.062 - 798.641 |
| Num Wives or Co-Wives | 0.586 | 0.263 - 1.303 | 0.263 | 0.008 - 8.715 |
| Total children | 5.099 | 1.010 - 25.757* | 3.923 | 0.743 - 20.712 |
| Total cattle | 0.986 | 0.963 - 1.009 | 0.978 | 0.952 - 1.005 |
| Wealth | 0.452 | 0.158 - 1.294 | 1.797 | 0.032 - 100.035 |
| AMR Knowledge | 1.017 | 0.310 - 3.335 | 0.028 | 0.001 - 0.748* |
| Bacteria Knowledge | 2.685 | 0.847 - 8.510 | 1.460 | 0.208 - 10.247 |
| Pseudo R2 | 0.32 |  | 0.65 |  |
| Prob>chi2 | 0.08 |  | 0.01 |  |
| Log Likelihood | -15.38 |  | -5.16 |  |
| LR chi2 | 14.24 |  | 19.45 |  |
| Obs | 40 |  | 22 |  |

**Table S10. Correlates of Innovation Use in Women and Men.** Reduced models using stepwise backward regression (0.20 retention factor).

|  | Women |  | Men |  |
| --- | --- | --- | --- | --- |
|  | B | SE | B | SE |
| Age | 0.864 | 0.755 - 0.989* | 0.856 | 0.692 - 1.058 |
| Bacteria Knowledge | 1.882 | 0.795 - 4.455 |  |  |
| Wealth | 0.441 | 0.165 - 1.174 |  |  |
| Total children | 2.332 | 1.115 - 4.877* | 1.247 | 0.985 - 1.578 |
| AMR Knowledge |  |  | 0.089 | 0.009 - 0.901* |
| Pseudo R2 | 0.24 |  | 0.43 |  |
| Prob>chi2 | 0.03 |  | 0.01 |  |
| Log Likelihood | -17.16 |  | -8.54 |  |
| LR chi2 | 10.66 |  | 12.68 |  |
| Obs | 40 |  | 22 |  |

**Figure 17. Fit Statistics Comparing Full and Reduced Models for Correlates of Innovation Use in Women.** Reduced model indices are in “Current” column and Full in “Saved” column.


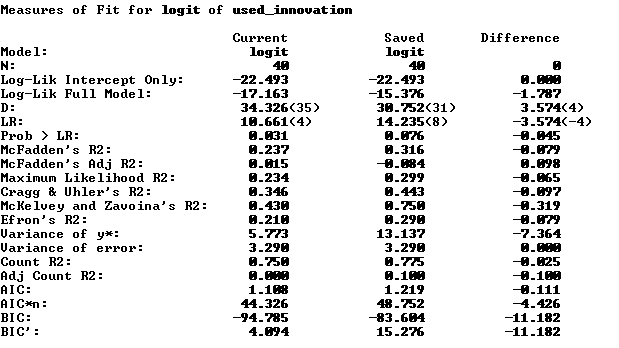


**Figure 18. Collinearity Statistics for Number of Steps Performed Correctly in Women.**


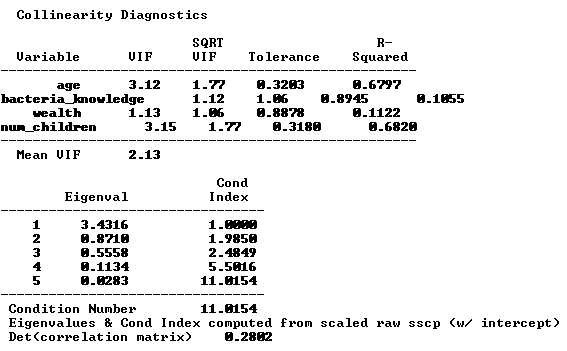


**Figure 19. Fit Statistics Comparing Full and Reduced Models for Correlates of Innovation Use in Men.** Reduced model indices are in “Current” column and Full in “Saved” column.


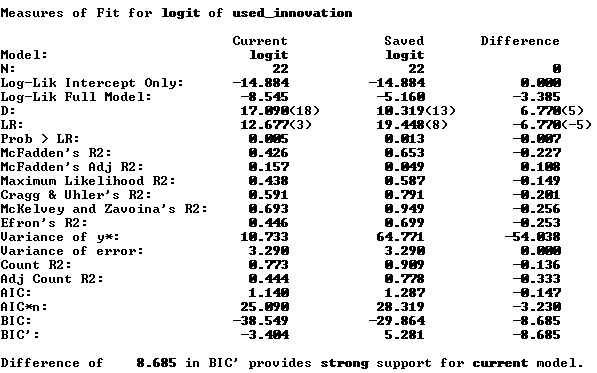


**Figure 20. Collinearity Statistics for Number of Steps Performed Correctly in Women.**


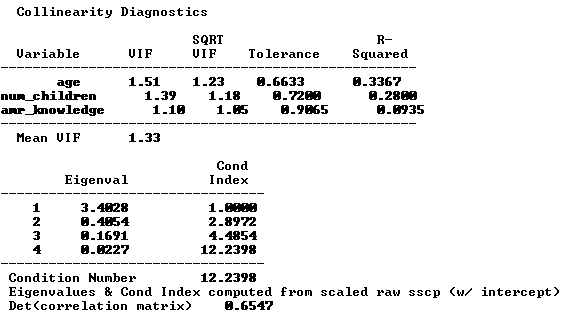

Supplement: Supplementary file 3 — Full Poisson and Logit Models, with Model fit Indices and Variance Inflation Factors (VIFs) for all Models Full models include: Poisson Models of the number of bacteria (Table S1) and AMR (Table S2) knowledge items recalled versus demographic and socioeconomic variables, for all participants combined, women, and men; Poisson models of the number of health benefit knowledge items recalled (Table S3) and the number of innovation use steps performed correctly (Table S4) versus demographic and socioeconomic variables, by gender; and a logit model of whether an innovation was used (1) or not (0) versus demographic and socioeconomic variables, by gender (Table S5). (DOCX 307 kb) [file 12879_2017_2857_MOESM3_ESM.docx]
